# Computational Approach Reveals Pronociceptive Potential of Cannabidiol in Osteoarthritis: Role of Transient Receptor Potential Channels

**DOI:** 10.3390/ph14100964

**Published:** 2021-09-24

**Authors:** Jakub Mlost, Marta Kędziora, Katarzyna Starowicz

**Affiliations:** Department of Neurochemistry, Maj Institute of Pharmacology, Polish Academy of Sciences, Smętna 12, 31-343 Cracow, Poland; mlost@if-pan.krakow.pl (J.M.); bryk@if-pan.krakow.pl (M.K.)

**Keywords:** cannabidiol, osteoarthritis, chronic pain, neuropathic pain, systems pharmacology

## Abstract

Systems pharmacology employs computational and mathematical methods to study the network of interactions a drug may have within complex biological pathways. These tools are well suited for research on multitarget drugs, such as natural compounds, in diseases with complex etiologies, such as osteoarthritis (OA). The present study focuses on cannabidiol (CBD), a non-psychoactive constituent of cannabis, targeting over 60 distinct molecular targets as a potential treatment for OA, a degenerative joint disease leading to chronic pain with a neuropathic component. We successfully identified molecular targets of CBD that were relevant in the context of OA treatment with both beneficial and detrimental effects. Our findings were confirmed by in vivo and molecular studies. A key role of PPARγ in mediating the therapeutic potential of CBD was revealed, whereas upregulation of multiple transient receptor potential channels demasked CBD-induced heat hyperalgesia. Our findings pave the way for novel CBD-based therapy with improved therapeutic potential but also encourage the use of bioinformatic tools to predict the mechanism of action of CBD in different conditions. We have also created an accessible web tool for analogous analysis of CBD pharmacology in the context of any disease of interest and made it publicly available.

## 1. Introduction

Systems pharmacology is a novel biological discipline employing computational and mathematical analysis and modelling to understand how drugs affect complex biological systems. In contrast to a reductionist perception of drug effect as a result of one specific drug–protein interaction, systems pharmacology considers the effect of a drug to be the outcome of the network of possible interactions. These tools are well suited to analyze and predict the outcome of multitarget drugs, such as natural compounds, especially in diseases with complex etiology. In the present study, we employed a systems pharmacology approach to elucidate the therapeutic potential and mechanism of action of cannabidiol (CBD) in osteoarthritis (OA).

Compelling evidence suggests active participation of the endocannabinoid system in the pathophysiology of joint pain associated with OA. To date, numerous studies have revealed beneficial effects of cannabinoids in preclinical models of OA [1]. This pool of studies involves a multitarget approach [2] as well as CB2 agonism, which has been shown to possess not only antinociceptive and anti-inflammatory effects but also the ability to slow OA progression [3].

The present study focuses on CBD, a non-psychoactive constituent of cannabis, which is characterized by complex pharmacology with greater than 60 distinct molecular targets [4]. We employed systems pharmacology tools to investigate the therapeutic potential of CBD, particularly in the context of the difficult-to-treat neuropathic component of OA. We combined publicly available databases such as STRING and OpenTargets to create a network of proteins involved in the specific disease in order to analyze pharmacodynamic properties of CBD and its therapeutic potential within the given context. This bioinformatic approach successfully identified molecular targets of CBD that were relevant in the context of OA treatment, and these targets were classified as both beneficial and detrimental. For example, CBD activates multiple transient receptor potential (TRP) channels, which are a group of cation permeable ion channels involved in pain signaling and their upregulation and sensitization was implicated as a key underlying factor of neuropathic components in OA [2], as well as the PPARγ receptor, which is a nuclear receptor mediating anti-inflammatory properties of cannabinoids [5,6]. Indeed, our experimental approach was able to discern the specific role of each receptor type in both pro and antinociceptive potential of CBD in the animal model of OA induced by sodium monoiodoacetate (MIA). Moreover, the physiological relevance of our findings was further confirmed by molecular studies. Thus, we have also created an accessible web tool for analogous analysis of CBD pharmacology in the context of any disease of interest and made it publicly available at https://jakub-mlost.shinyapps.io/CBDpharm/ (accessed on 21 September 2021) and GitHub.

## 2. Results

### 2.1. Identification of Potential Therapeutic Targets of CBD of Neuropathic Component in Osteoarthritis

**Figure 1:**
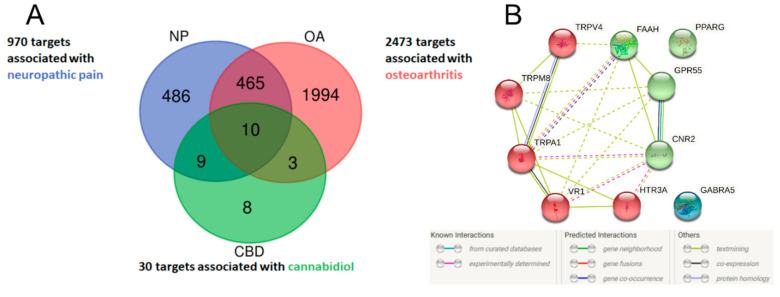
Venn diagram of potential therapeutic targets of CBD in the neuropathic component of OA (**A**). Lists of disease-associated targets were obtained from the Open Targets Platform, and a list of CBD targets was created based on a literature search. Ten CBD targets associated with neuropathic pain and osteoarthritis were visualized in STRING and clustered into either ionotropic receptors or proteins associated with the endocannabinoid system (**B**).

**Figure 2:**
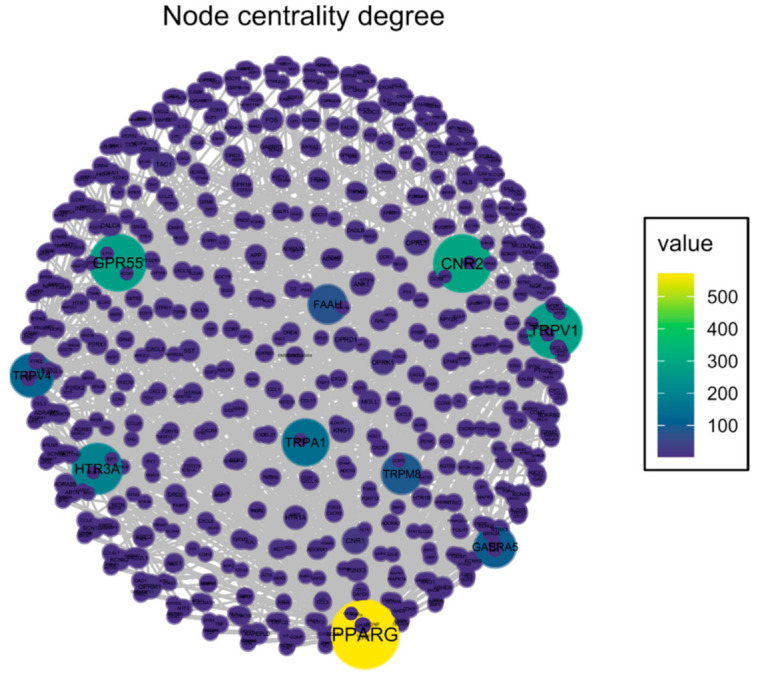
Graph presenting CBD target interactions with other proteins associated with osteoarthritis and neuropathic pain. Node color represents the number of interactions for a specific protein (vertex degree). Network was visualized with the “Influential” package in R.

An Open Targets Platform search identified 2473 unique molecular targets associated with OA and 970 targets associated with neuropathic pain, of which 475 targets were associated with both conditions (Figure 1). CBD has been reported to interact with 66 targets in the literature; however, many of those interactions were studied in concentrations well beyond physiologically relevant levels. Thus, for further analysis, we limited the list of CBD targets to 30 targets with Ki or EC/IC_50_ values < 2 µM. We found 10 targets of CBD to be associated with both neuropathic pain and OA (Figure 1A). These targets were analyzed, clustered, and visualized with the protein–protein interaction database service STRING (Figure 1B). We were able to distinguish CBD targets as either ionotropic receptors permeable for cations or anions as well as proteins involved in endocannabinoid system functioning (Figure 1B). Functional enrichment analysis of biological processes revealed those proteins to be strongly involved in thermoception and pain (Table 1). Finally, we performed an analysis of the protein–protein interaction of CBD targets with proteins associated with OA and neuropathic pain. We found that among CBD targets, the PPARγ receptor interacts with the highest number of other proteins associated with both pathological conditions (Figure 2 and Table 2).

### 2.2. In Vivo Assessment of CBD Efficacy in an Animal Model of OA

**Figure 3:**
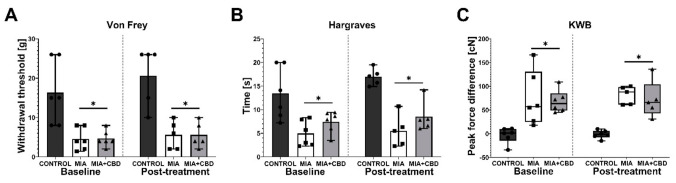
Analgesic potential of 10 mg/kg CBD at Day 20 and 21 post MIA induction. Baseline assessment was performed at Day 20 post MIA, whereas post-treatment assessment was performed 2 h after i.p. administration of CBD (10 mg/kg) (**A**–**C**). Two-way ANOVA identified treatment as significant source of variation in all tests (*p* < 0.0001). Individual data points are shown in box and whisker plots presenting the means ± min to max. ● denotes samples in control group; ■ denotes samples in MIA group; ▲ denotes samples in MIA + CBD group. Each experimental group included N = 5–6 rats. The difference was considered significant when *p* < 0.05. * Denotes significant differences compared with the vehicle-treated control group of healthy animals.

We first studied the dose of 10 mg/kg CBD at Day 21 post OA induction. Baseline measurements revealed significant mechanical and thermal hypersensitivity in the von Frey and Hargreaves tests (Figure 3A,B), as noted by a decrease in both the paw withdrawal threshold (Figure 3A) and latency to pain response (Figure 3B). Moreover, MIA administration induced weight bearing impairment in the KWB assessment, which manifested as an increase in discrepancy between forces applied by the rear left and rear right paws (Figure 3C). Administration of 10 mg/kg CBD did not significantly affect these changes (Figure 3A–C) when measured 2 h post i.p. injection.

**Figure 4:**
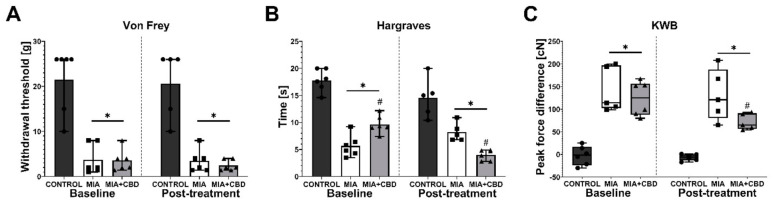
Analgesic potential of 50 mg/kg CBD at Day 27 and 28 post MIA induction. Assessment was performed after a 6-day wash-out period on the same rats presented in Figure 4. Pain was assessed at baseline at Day 27 post MIA (Baseline) and 2 h after i.p. administration of CBD (50 mg/kg) at Day 28 post MIA (Post-treatment) (**A**–**C**). Two-way ANOVA revealed significant treatment effect in all tests (*p* < 0.0001). Individual data points are shown in box and whisker plots presenting the means ± min to max. Each experimental group included N = 5–6 rats. ● denotes samples in control group; ■ denotes samples in MIA group; ▲ denotes samples in MIA + CBD group. The difference was considered significant when *p* < 0.05. * Denotes significant differences compared with the vehicle-treated control group of healthy animals; # denotes difference compared with the vehicle-treated MIA group.

After a 6-day wash-out period, the same rats were administered i.p. with vehicle or 50 mg/kg CBD. Similarly, at baseline recordings, we observed mechanical allodynia and weight bearing impairment in MIA rats (Figure 4A,C). However, the analysis of baseline measurements revealed a decrease in heat hyperalgesia measured in the Hargreaves test in rats that had previously received 10 mg/kg CBD (Figure 4B). Interestingly, administration of 50 mg/kg CBD did not influence mechanical allodynia (Figure 4A). However, this dose exacerbated heat hyperalgesia compared to vehicle-treated MIA rats (Figure 4B) but decreased the difference in hind paw weight bearing (Figure 4C). These results suggest both a decrease in pain perception of freely moving animals and an increase in sensitivity to thermal stimuli but not mechanical stimuli.

### 2.3. Establishing CBD’s Mechanism of Action in Animal Model of OA

**Figure 5:**
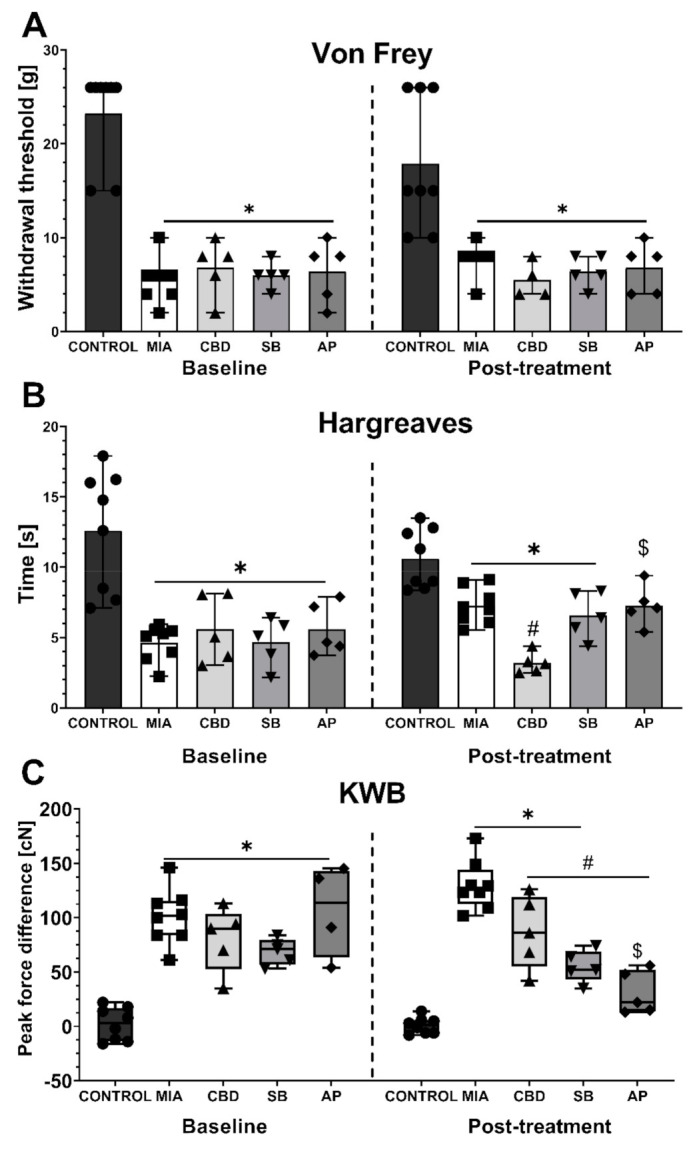
Analgesic potential of 50 mg/kg CBD at Day 20 and 21 post MIA induction modulated by coadministration of TRPV1 and TRPA1 antagonists. CBD was administered i.p. two hours before assessment. The TRPV1 antagonist SB-366791 (SB; 1 mg/kg) and TRPA1 antagonist AP-18 (AP; 0.2 mg/kg) were administered i.p. 30 min before the assessment. Pain was assessed at baseline at day 20 post MIA and 2 h after i.p. administration of CBD with or without antagonists at day 21 post MIA (**A**–**C**). Two-way ANOVA revealed significant treatment effect in all tests (*p* < 0.0001). Individual data points are shown in box and whisker plots presenting means ± min to max. The control and MIA groups included N = 8 rats, whereas the CBD group without antagonists (CBD, SB, AP) included N = 5 rats. ● denotes samples in control group; ■ denotes samples in MIA group; ▲ denotes samples in MIA + CBD group; ▼ denotes samples in MIA + CBD + SB-366791 group; ♦ denotes samples in MIA + CBD + AP-18 group. The difference was considered significant when *p* < 0.05. * Denotes significant differences compared with the vehicle-treated control group of healthy animals; # denotes difference compared with the vehicle-treated MIA group; $ denotes difference compared with the CBD group.

To establish the mechanism of the pro- and antinociceptive action of CBD, we have followed on from the information presented in Table 2. We decided to focus only on targets activated by CBD so their role could be easily revealed by specific antagonistic compounds. Thus, the TRPV1 antagonist SB-366791 (SB; 1 mg/kg), TRPA1 antagonist AP-18 (AP; 0.2 mg/kg), TRPV4 antagonist HC-067047 (HC; 10 mg/kg), and PPARγ antagonist GW9662 (GW; 2 mg/kg) were tested in combination with 50 mg/kg CBD. First, at Day 20 post MIA injection, we performed baseline measurements, which revealed mechanical allodynia (Figure 5A), hot hypersensitivity (Figure 5B), and weight bearing impairment (Figure 5C) in all groups following MIA injection compared to the control group. No difference was observed among the MIA-treated groups (Figure 5A–C) at baseline. CBD (50 mg/kg) administration for 2 h before the assessment at Day 21 post MIA injection did not affect mechanical allodynia (Figure 5A) compared to the vehicle-treated MIA group. Similar to that noted in a previous experiment, CBD (50 mg/kg) exacerbated heat hypersensitivity in OA animals compared to vehicle-treated MIA rats (Figure 5B) but improved weight bearing discrepancies in KWB measurements (Figure 5C). The TRPV1 antagonist SB-366791 and the TRPA1 antagonist AP-18 were administered 30 min before the assessment of CBD effects in OA animals (SB and AP, respectively). SB did not modulate mechanical allodynia (Figure 5A) or weight bearing impairment (Figure 5C) in CBD-treated OA animals, although its effects showed a decreasing trend in the hyperalgesic phenotype (Figure 5B). TRPA1 antagonist coadministration (AP) did not affect mechanical hypersensitivity in CBD-treated MIA animals (Figure 5A). However, AP-18 significantly reduced heat hypersensitivity (Figure 5B) and restored normal weight bearing (Figure 5C) in CBD-treated MIA animals compared to CBD-treated MIA animals that did not receive AP-18 (CBD vs. AP).

**Figure 6:**
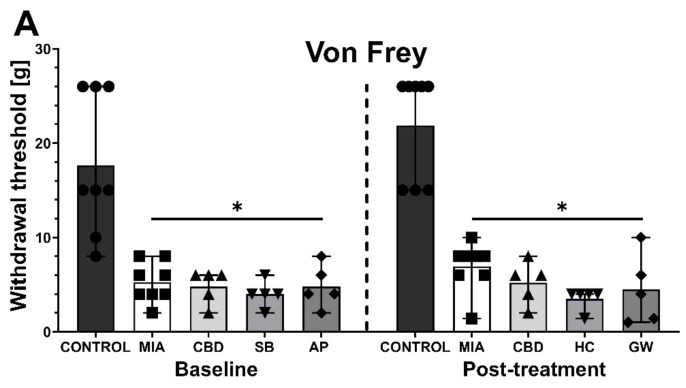

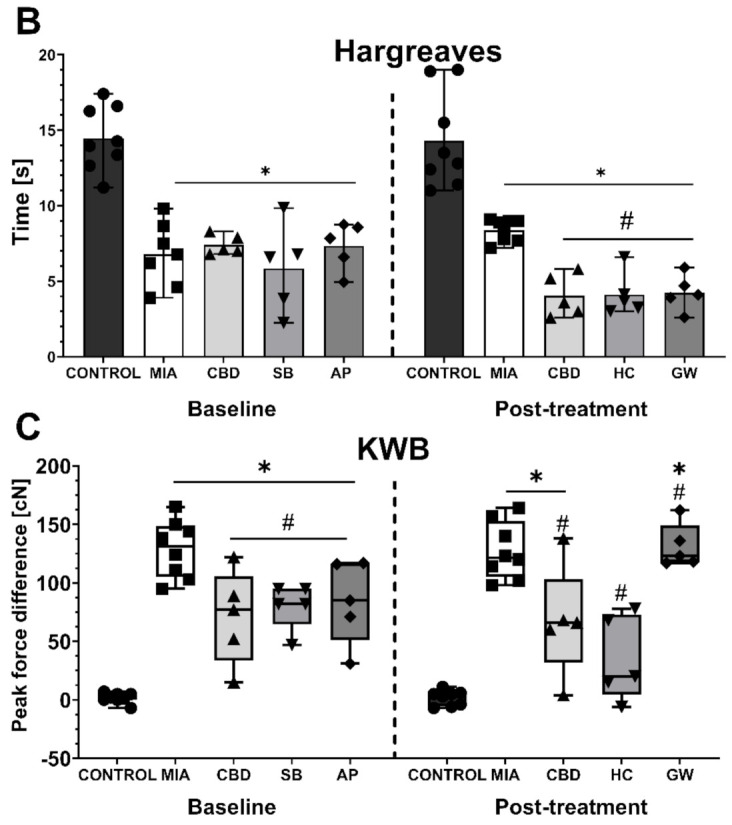
Analgesic potential of 50 mg/kg CBD at Day 27 and 28 post MIA induction modulated by coadministration of TRPV4 and PPARγ antagonists. Assessment was performed after a 6-day wash-out period on the same rats presented in Figure 5, which were coadministered CBD (50 mg/kg) with SB-366791 (SB; 1 mg/kg) and the TRPA1 antagonist AP-18 (AP; 0.2 mg/kg). CBD alone or in combination with the PPARγ antagonist GW9662 (GW, 2 mg/kg) was administered i.p. Two hours before assessment, the TRPV4 antagonist HC-067047 (HC, 10 mg/kg) was administered i.p. 30 min before the assessment. Pain was assessed at baseline at day 27 post MIA and 2 h after i.p. administration of CBD with or without antagonists at day 28 post MIA (**A**–**C**). Two-way ANOVA revealed significant treatment effect in all tests (*p* < 0.0001). Individual data points are shown in box and whisker plots presenting means ± min to max. The control and MIA groups included N = 8 rats, whereas the CBD group without antagonists (CBD, AP/HC, SB/GW) included N = 5 rats. ● denotes samples in control group; ■ denotes samples in MIA group; ▲ denotes samples in MIA + CBD group; ▼ denotes samples in MIA + CBD + SB-366791 or HC-067047 group; ♦ denotes samples in MIA + CBD + AP-18 or GW9662 group. The difference was considered significant when *p* < 0.05. * Denotes significant differences compared with the vehicle-treated control group of healthy animals; # denotes difference compared with the vehicle-treated MIA group; $ denotes difference compared with the CBD group.

After a 6-day wash-out period, we performed subsequent baseline measurements before the administration of the PPARγ antagonist GW9662 (GW; 2 mg/kg) or the TRPV4 antagonist HC-067047 (HC; 10 mg/kg) in combination with CBD (50 mg/kg). GW9662 was administered simultaneously with CBD for 2 h before the behavioral assessment, whereas HC-067047 was administered after CBD injection for 30 min before the assessment at Day 28 post MIA injection. HC-067047 was administered to rats that had previously received AP-18, whereas GW9662 was administered to rats that had previously received SB-366791. At baseline at Day 27 post MIA injection, we observed a significant increase in mechanical and heat hypersensitivity as well as weight bearing impairment in MIA-treated animals (Figure 6A–C). Moreover, we observed a significant improvement in weight bearing at Day 27 following previous administration of CBD alone or in combination with SB-366791 or AP-18 at Day 21 compared to vehicle-treated MIA animals (Figure 6C). No effects of previous pharmacological treatment were observed in the case of mechanical and heat hypersensitivity (Figure 6B,C). Similar to previous experiments, CBD administration did not affect mechanical hypersensitivity (Figure 6A). However, CBD exacerbated heat hypersensitivity (Figure 6B) while improving the balance in weight bearing (Figure 6C) in OA animals compared to vehicle-treated MIA rats (MIA vs. CBD). HC-067047 coadministration did not modulate CBD effects upon thermal and mechanical hypersensitivity (Figure 6A,B); however, it restored weight bearing to a point that was no longer significantly different from healthy control animals (Figure 6C). In contrast to the TRP antagonists, coadministration of the PPARγ antagonist GW9662 blocked the beneficial effects of CBD on weight bearing (Figure 6C) but did not affect mechanical or heat hypersensitivity (Figure 6A,B).

### 2.4. CBD’s Molecular Mechanism of Action within the Lumbar Spinal Cord in an Animal Model of OA

**Figure 7:**
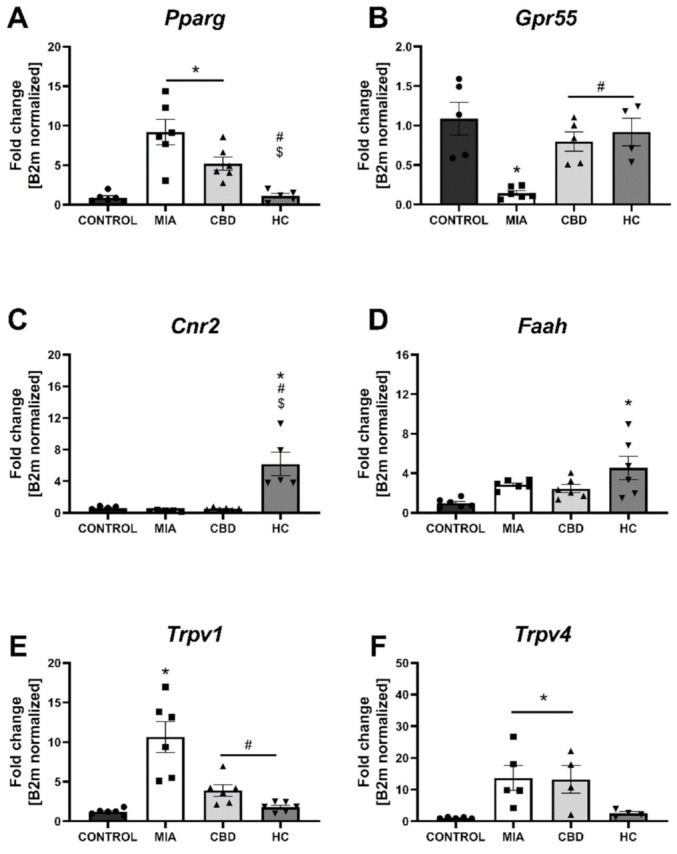

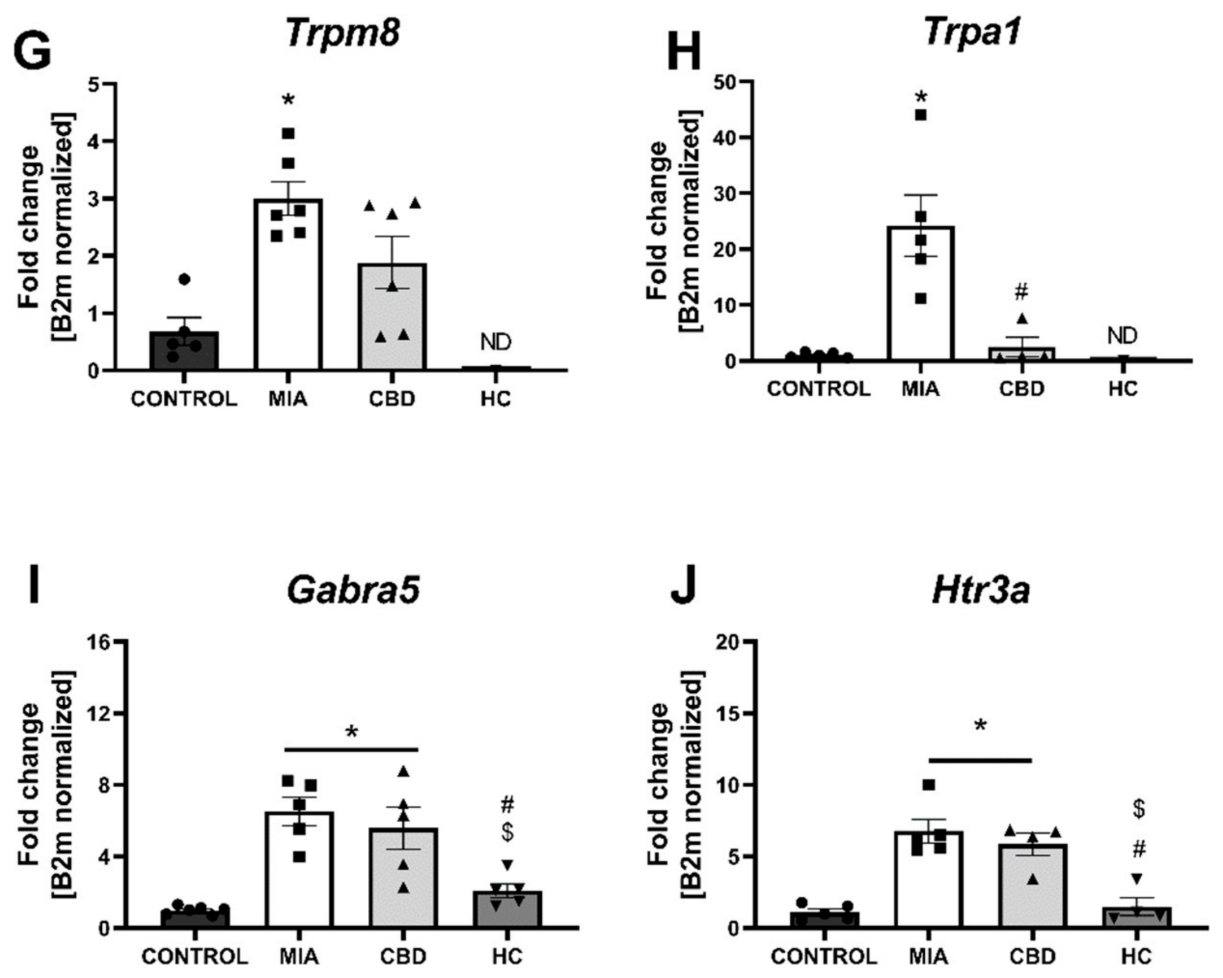
Molecular changes in CBD-associated target expression in the lumbar spinal cord. Gene expression of *Pparg* (**A**), *Gpr55* (**B**), *Cnr2* (**C**), *Faah* (**D**), *Trpv1* (**E**), *Trpv4* (**F**), *Trpm8* (**G**), *Trpa1* (**H**), *Garba5* (**I**) and *Htr3a* (**J**) was evaluated in the lumbar spinal cord at Day 28 post MIA and 5 h after drug administration. CBD was administered at a dose of 50 mg/kg at both Days 21 and 28 post MIA injection either alone (CBD) or in combination with AP-18 (0.2 mg/kg) at Day 21 and HC-067047 (10 mg/kg) at Day 28 post MIA (HC). ANOVA revealed significant changes in all tested parameters with *p* < 0.05. Individual data points are shown in boxes presenting the mean ± SEM of fold change normalized to the reference gene beta-2 microglobulin (*B2m*). ● denotes samples in control group; ■ denotes samples in MIA group; ▲ denotes samples in MIA + CBD group; ▼ denotes samples in MIA + CBD + AP-18 followed by HC-067047 group. Statistical analysis was performed using one-way ANOVA followed by Tukey’s post hoc test with a *p* < 0.05 confidence interval. Each experimental group includes N = 4–6 rats. * Denotes significant differences vs. control; # Denotes significant differences vs. MIA; $ Denotes significant differences vs. CBD.

**Figure 8:**
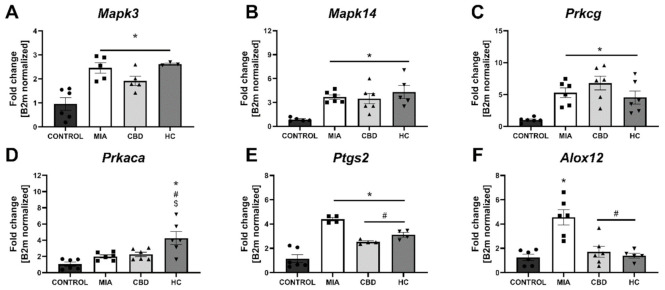
Molecular changes in CBD-associated target gene expression in the lumbar spinal cord. Gene expression of *Mapk3* (**A**), *Mapk14* (**B**), *Prkcg* (**C**), *Prkaca* (**D**), *Ptgs2* (**E**) and *Alox12* (**F**) was evaluated in the lumbar spinal cord at Day 28 post MIA and 5 h after drug administration. CBD was administered at a dose of 50 mg/kg at both Days 21 and 28 post MIA injection either alone (CBD) or in combination with AP-18 (0.2 mg/kg) at Day 21 and HC-067047 (10 mg/kg) at Day 28 post MIA (HC). ANOVA revealed significant changes in all tested parameters with *p* < 0.05. Individual data points are shown in boxes presenting the mean ± SEM of fold change normalized to the reference gene beta-2 microglobulin (*B2m*). ● denotes samples in control group; ■ denotes samples in MIA group; ▲ denotes samples in MIA + CBD group; ▼ denotes samples in MIA + CBD + AP-18 followed by HC-067047 group. Statistical analysis was performed using one-way ANOVA followed by Tukey’s post hoc test with a *p* < 0.05 confidence interval. Each experimental group includes N = 4–6 rats. * Denotes significant differences vs. control; # Denotes significant differences vs. MIA; $ Denotes significant differences vs. CBD.

We performed RT–qPCR analysis of CBD-associated targets with OA and neuropathic pain (identified in Section 2.1) to assess the expression of all the identified CBD-associated targets in the lumbar spinal cord of both control and MIA animals (Figure 7). MIA administration caused a significant increase in the expression of *Pparg* (Figure 7A) and a decrease in *Gpr55* expression (Figure 7B) together with a robust upregulation of genes coding for ionotropic receptors, including *Trpv1*, *Trpv4*, *Trpm8*, *Trpa1*, *Gabra5*, and *Htr3a* (Figure 7E–J). No changes in *Cnr2* and *Faah* expression were detected in MIA and control animals, but these genes were significantly upregulated following CBD cotreatment with transient receptor potential (TRP) channel antagonists (Figure 7C,D). Moreover, CBD treatment restored *Gpr55* expression (Figure 7B) and reduced *Trpv1* (Figure 7E), *Trpm8*, and *Trpa1* expression (Figure 7G,H). In addition, coadministration of TRP antagonists along with CBD further reduced the expression of *Pparg* (Figure 7A), *Trpv4*, *Trpm8*, *Trpa1*, *Gabra5*, and *Htr3a* (Figure 7F–J) in the lumbar spinal cord. Based on our previous paper assessing the role of the TRPV1 receptor in osteoarthritis [6], we decided to evaluate the expression of TRPV1-sensitizing factors in the lumbar spinal cord. In addition to CBD-associated targets, we observed upregulation of *Mapk3*, *Mapk14*, and *Prkcg* in the lumbar spinal cord of MIA animals (Figure 8A–C). These changes were accompanied by increased levels of *Ptgs2* and *Alox12* transcripts (Figure 8E,F). CBD treatment did not affect the expression of *Mapk3*, *Mapk4*, and *Prkcg* (Figure 8A–C) but reduced the expression of the proinflammatory factors *Ptgs2* and *Alox12* (Figure 8E,F). Prkaca expression was not affected by either MIA or CBD, but it was increased following TRP channel antagonist cotreatment with CBD (Figure 8D).

### 2.5. Establishing CBD’s Molecular Mechanism of Action within Cartilage and Subchondral Bone in an Animal Model of OA

**Figure 9:**
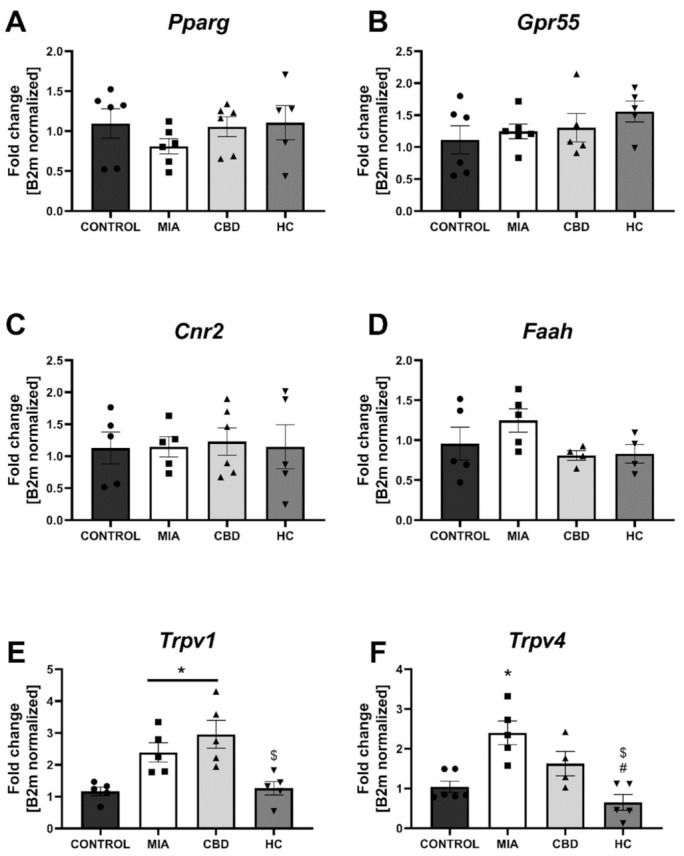

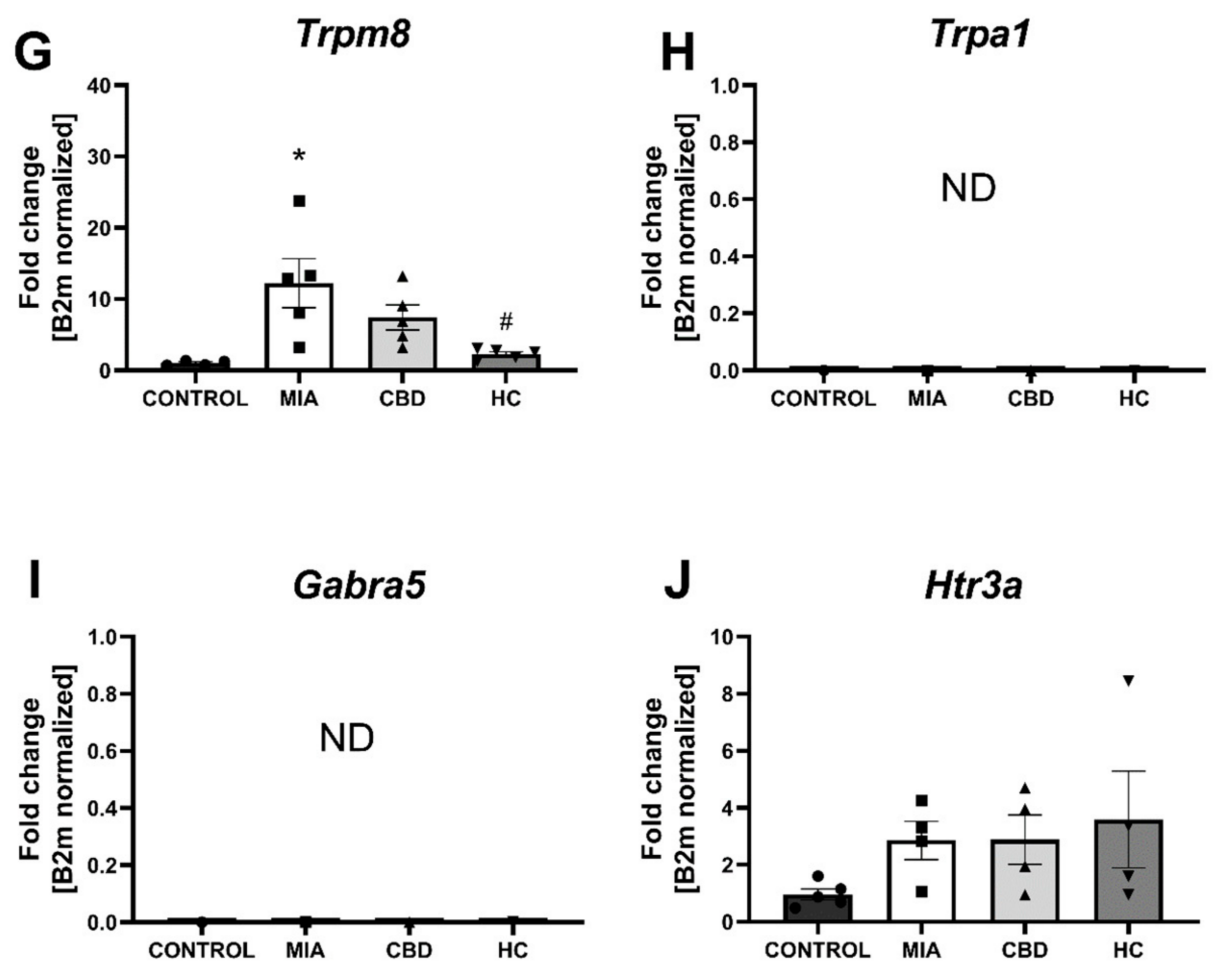
Molecular changes in CBD-associated target expression in rat cartilage and subchondral bone. Expression of *Pparg* (**A**), *Gpr55* (**B**), *Cnr2* (**C**), *Faah* (**D**), *Trpv1* (**E**), *Trpv4* (**F**), *Trpm8* (**G**), *Trpa1* (**H**), *Garba5* (**I**) and *Htr3a* (**J**) was evaluated in cartilage and subchondral bone tissue at Day 28 post MIA and 5 h after drug administration. CBD was administered at a dose of 50 mg/kg at both Days 21 and 28 post MIA injection either alone (CBD) or in combination with AP-18 (0.2 mg/kg) at Day 21 and HC-067047 (10 mg/kg) at Day 28 post MIA (HC). ANOVA revealed significant changes in expression of *Trpv1*, *Trpv4*, and *Trpm8* with *p* < 0.01. Individual data points are shown in boxes presenting the mean ± SEM of fold change normalized to the reference gene beta-2 microglobulin (*B2m*). ● denotes samples in control group; ■ denotes samples in MIA group; ▲ denotes samples in MIA + CBD group; ▼ denotes samples in MIA + CBD + AP-18 followed by HC-067047 group. Statistical analysis was performed using one-way ANOVA followed by Tukey’s post hoc test with a *p* < 0.05 confidence interval. Each experimental group includes N = 4–6 rats. * Denotes significant differences vs. control; # Denotes significant differences vs. MIA; $ Denotes significant differences vs. CBD.

**Figure 10:**
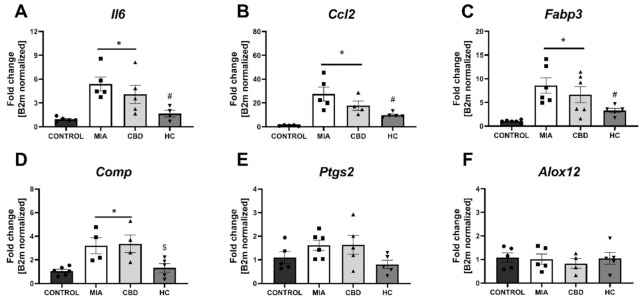
Molecular changes in inflammatory factor expression in rat cartilage and subchondral bone. Gene expression of *Il6* (**A**), *Ccl2* (**B**), *Fabp3* (**C**), *Comp* (**D**), *Ptgs2* (**E**) and *Alox12* (**F**) was evaluated in cartilage and subchondral bone tissue at Day 28 post MIA and 5 h after drug administration. CBD was administered at a dose of 50 mg/kg at both Days 21 and 28 post MIA injection, either alone (CBD) or in combination with AP-18 (0.2 mg/kg) at Day 21 and HC-067047 (10 mg/kg) at Day 28 post MIA (HC). ANOVA revealed significant changes in expression of *Il6*, *Ccl2*, *Fabp3*, *Comp* with *p* < 0.005. Individual data points are shown in boxes presenting the mean ± SEM of fold change normalized to the reference gene beta-2 microglobulin (*B2m*). ● denotes samples in control group; ■ denotes samples in MIA group; ▲ denotes samples in MIA + CBD group; ▼ denotes samples in MIA + CBD + AP-18 followed by HC-067047 group. Statistical analysis was performed using one-way ANOVA followed by Tukey’s post hoc test with a *p* < 0.05 confidence interval. Each experimental group includes N = 4–6 rats. * Denotes significant differences vs. control; # Denotes significant differences vs. MIA; $ Denotes significant differences vs. CBD.

In cartilage and subchondral bone, we did not observe changes in the expression of *Pparg*, *Gpr55*, *Cnr2*, *Faah* (Figure 9A–D), and *Htr3a* (Figure 9J) between groups, but significant upregulation of *Trpv1*, *Trpv4*, and *Trpm8* was observed in MIA animals (Figure 9E–G). *Trpa1* and *Gabra5* expression levels in the cartilage and subchondral bone were below the detection level in all groups (Figure 9H,I). CBD treatment partially abolished *Trpv4* and *Trpm8* upregulation in MIA animals, but only cotreatment with the TRP antagonist significantly decreased the expression of all detected TRP channels (Figure 9E–H). Based on our paper reporting molecular changes in the cartilage and subchondral bone following CB2 activation [3], we decided to evaluate CBD’s effects on previously described genes. Similar to previous findings, we observed an upregulation of *Il6*, *Ccl2*, *Fabp3*, and *Comp* but not *Ptgs2* and *Alox12* (Figure 10A–F) in MIA cartilage. CBD treatment only was not able to reduce expression of the upregulated genes (Figure 10A–D); however, CBD cotreatment with a TRP antagonist decreased the expression of *Il6*, *Ccl2*, *Fabp3*, and *Comp* compared to MIA animals (Figure 10A–D).

## 3. Discussion

Herein, the presented results describe a bioinformatic approach for the assessment of the therapeutic potential of CBD and its mechanism of action in a disease with complex etiology. We identified up to 10 distinct physiological targets of CBD that are implicated in both OA and neuropathic pain. In fact, those 10 targets were mostly involved in thermoception, calcium transmembrane transport, and pain. Further network analysis revealed PPARγ, CB2, and GPR55 as the three most targetable proteins for OA management, and a significant proportion of TRP channels were identified as potential modulators that could mask the therapeutic potential of CBD. Indeed, pharmacological studies of CBD in vivo allowed us to discern the bidirectional impact of CBD upon pain sensation in OA animals. Our data showed that CBD administration restored impaired weight bearing in OA animals, but CBD simultaneously exacerbated heat hyperalgesia. Consistent with our predictions, we were able to block CBD-induced weight bearing restoration by a PPARγ antagonist, while CBD-induced heat hyperalgesia was blocked by TRPV1 or TRPA1 antagonists but not a TRPV4 antagonist. It is important to mention the slight advantage of TRPA1 over TRPV1 antagonism in potentiating the analgesic potential of CBD. Moreover, CBD alone was not able to modulate mechanical allodynia in the von Frey test or cold hyperalgesia of the ipsilateral osteoarthritic paw, suggesting that restoration of weight bearing is due to anti-inflammatory action rather than direct antinociceptive effects. Regarding the anti-inflammatory potential of CBD, we also observed significantly improved pain behavior after a 7-day wash-out period at Day 28 post MIA, which suggests prolonged improvement in the OA phenotype. We observed similar effects following acute treatment with a CB2 agonist (data not published). However, this effect was dose and receptor dependent given that the ineffective per se dose of 10 mg/kg CBD reduced heat hyperalgesia at Day 21 post MIA but failed to restore impaired weight bearing. In contrast, the effective dose of 50 mg/kg improved weight bearing at Day 28 post MIA but failed to alter heat hyperalgesia.

Quantitative assessment of gene expression adds some insight into the molecular explanation of the observed phenomena. We observed the expression of all physiologically relevant CBD targets in the lumbar spinal cord and a substantial increase in the expression of PPARγ and ionotropic receptors, including all assayed TRP channels and GABA receptor subunit alpha 5 and 5-HT3R. PPARγ upregulation may reflect ongoing inflammation, whereas upregulation of cationic TRP channels and 5-HT3R attest to sensitization of pain pathways as they are all involved in conveying excitatory pain signals through the spinal cord. CBD only reduced TRPV1 and TRPA1 expression, whereas CBD cotreatment with TRP antagonists reduced the expression of all ionotropic receptors. Interestingly, CBD cotreatment with a TRP antagonist increased endocannabinoid signaling through upregulation of CB2 and FAAH. Moreover, we observed a pronounced downregulation of GPR55 in the lumbar spinal cord of MIA animals that was reversed by CBD treatment with or without TRP antagonists. However, the physiological significance of these findings is difficult to explain given that little is known about the physiological function of GPR55 in the spinal cord, and knockout studies in pain models have yielded conflicting results [12,13]. It is plausible to suspect that some of the therapeutic potential of CBD is mediated through GPR55 antagonism given that its activation is related to calcium influx [8,14], but this hypothesis should be further studied in knockout animals. However, it is important to note that GPR55 downregulation in the MIA spinal cord was rather robust.

Similar to our previous findings [2], we observed significant upregulation of kinases involved in TRP channel sensitization in the lumbar spinal cord. CBD was not able to reverse this effect, although cotreatment with TRP antagonists further upregulated *Prkaca,* which codes for the catalytic subunit of protein kinase A. *Prkaca* expression was strongly correlated with *Faah* expression (r(19) = 0.88; *p* < 0.0001); therefore, it may reflect a decrease in endocannabinoid tone leading to an increase in the activity of protein kinase A. Moreover, upregulation of genes coding for enzymes involved in the production of proinflammatory mediators from arachidonic acid and anandamide, *Ptgs2* and *Alox12*, which are known causes of neuronal hyperexcitability, was observed in the lumbar spinal cord [15,16]. Importantly, CBD treatment decreased the expression of *Ptgs2* and *Alox12* in the lumbar spinal cord of MIA animals.

Although CBD activation of TRP channels is well described in the literature [9], the upregulation and sensitization of TRP channels in an animal model of osteoarthritis may explain why CBD-induced heat hyperalgesia was not observed before and also why CBD failed to meet the desired endpoint in clinical settings [17]. Of note, CBD possesses even more favorable antinociceptive molecular targets; the antinociceptive effects of 5-HTR3A antagonists have already been described [10,18,19], similar to positive allosteric modulation of the GABA_A_ receptor [11,20]. However, these effects may be masked through the activation of various TRP channels by CBD, as shown in the present study. Moreover, some of the CBD effects may also be mediated by CB2, the activation of which has been shown to exert beneficial effects in OA [3]. However, CBD activity toward CB2 is complicated with reports of partial agonism [7] and negative allosteric modulation [21]; thus, the interaction between CBD and CB2 in OA remains to be described as the effects may also be dependent on receptor availability [22].

In contrast to the spinal cord, the majority of CBD targets were not differentially expressed in the cartilage and subchondral bone of MIA rats. In fact, we were not even able to detect the expression of *Trpa1* and *Gabra5*. However, *Trpv1*, *Trpv4*, and *Trpm8* were not upregulated in MIA cartilage, and these levels were only partially reduced by CBD treatment but completely counteracted by CBD cotreatment with TRP antagonists. Although the physiological role of TRPV1 in cartilage is unclear, TRPV4 promotes cartilage extracellular matrix biosynthesis, upregulates pro anabolic and anticatabolic genes, and increases matrix accumulation [23,24,25]. However, studies have shown that cartilage-specific knockout of TRPV4 decreases age-related OA but not surgically induced OA [26]. Moreover, we examined the expression of some OA-associated targets in cartilage. CBD alone was not able to reduce the expression of upregulated genes; however, the combination with TRP antagonists significantly counteracted the upregulation of the proinflammatory factors IL6 and CCL2, the OA biomarker COMP, and the intracellular transporter of fatty acids FABP3. The underlying mechanism may be directly related to TRPV4 antagonism with HC-067047, as described by Xu et al., 2019. Briefly, TRPV4 activation due to mechanical load causes significant calcium influx, which triggers caspase-3/6/7/8 expression or upregulates the death domain of Fas-associated proteins, eventually leading to chondrocyte apoptosis or inhibition of proliferation. This effect may be blocked by TRPV4 antagonism [27]. Similarly, anti-inflammatory action combined with inhibition of neuronal excitability following CBD cotreatment with TRP channel antagonists could be responsible for the reversion of the OA-associated molecular phenotype in the spinal cord. However, more precise electrophysiological studies are needed to establish the role of each CBD target in conveying pain signaling.

## 4. Materials and Methods

### 4.1. Identification of Molecular Targets

Keywords “osteoarthritis” and “neuropathic pain” were used for searches in the Open Targets Platform (https://www.targetvalidation.org, accessed on 21 September 2021) to identify disease-associated targets. The Open Targets Platform is a comprehensive and authoritative database for screening and visualizing potential drug targets related to diseases; the platform integrates a variety of publicly available datasets to help users identify and prioritize targets for further research [28]. A database of CBD molecular targets was created based on a literature search for a review of CBD pharmacology [4] (data available as Table S1). Analysis was performed on targets with Ki or EC/IC_50_ values for CBD < 2 µM, which is the highest physiologically available concentration in humans [29]. Venn analysis was performed to isolate common targets among CBD, OA, and neuropathic pain using the “VennDiagram” package in R. Common targets were visualized using the STRING platform and analyzed for their involvement in biological processes using the Gene Ontology database with the whole genome as a background [30].

### 4.2. Construction of a Target–Target Interaction Network

A protein–protein interaction (PPI) network was created with the STRING database involving proteins associated with both OA and neuropathic pain. For further analysis, we isolated PPIs exclusively involving molecular targets of CBD. The vertex degree, a measure of centrality that indicates the number of connections of specific nodes within other nodes in the network, was calculated, and the network was visualized with the “Influential” package in R [31].

### 4.3. Animals

Male Wistar rats (Charles River, Hamburg, Germany) from approximately 55th postnatal day that initially weighed 225–250 g were used for all experiments. The animals were housed four per cage under a standard 12 h/12 h light/dark cycle with food and water available ad libitum. Animals were housed in conventional cages on aspen wood bedding without environmental enrichment. All experiments were approved by the Local Bioethics Committee of the Institute of Pharmacology (Cracow, Poland, approval number 308/2020, approval on 19 November 2020). All pharmacological experiments (including treatment and behavioral assays) were performed in the morning hours (08:00–12:00). Tissue dissection was performed at the end of the experiments. Care was taken to implement the “3 Rs” rule (replacement, reduction, and refinement) to reduce the number of animals used and their suffering during the experiments. Minimal number of animals was chosen based on our previous experience with MIA model of OA and experimental methods used herein. Due to largely reproducible phenotype of MIA animals and small drop-out rate in the following molecular analysis, we could use 6 animals per group in our experiments.

### 4.4. Drugs and Reagents

CBD was obtained from THC-pharm (Frankfurt am Main, Germany). GW9662, HC-067047, AP-18, SB-366791, MIA and Kolliphor EL were obtained from Sigma-Aldrich (Darmstadt, Germany). Drugs were dissolved in a vehicle solution containing 5% Kolliphor^®^ EL and 5% ethanol in 0.9% saline. MIA was dissolved in 0.9% saline.

### 4.5. OA Induction

Animals were deeply anaesthetized with 5% isoflurane in 100% O_2_ (3.5 L/min) in SomnoSuite^®^ (Kent Scientific Corporation, Torrington, CT, USA) until the flexor withdrawal reflex was abolished. The skin overlying the rear right knee joint was shaved and swabbed with 100% ethanol. A 27-gauge needle was introduced into the joint cavity through the patellar ligament, and 1 mg of MIA, which is an irreversible GAPDH inhibitor, diluted in 50 µL of 0.9% saline, was injected into the joint (intra-articular, i.a.) to induce OA-like lesions. MIA inhibits chondrocyte glycolysis and reproduces osteoarthritis-like histological lesions and functional impairment similar to that observed in human disease [32]; it is acclaimed for a consistent and reproducible pain phenotype suitable for research on analgesic drugs [33,34,35]. The age and weight of the animals were selected to allow comfortable access for i.a. injection.

### 4.6. Treatment Paradigm

To reduce the number of animals used in the experiments, we performed two behavioral assays on one set of animals with a 7-day wash-out period at Days 21 and 28 post MIA induction, as both timepoints are characterized by maximum cartilage damage and a pain phenotype in the given model of OA [36]. CBD doses were selected particularly on effective doses reported by Xiong et al., 2012 [37], while antagonists doses, cotreatment schedules, and behavioral assessment timepoints were based on the literature [4,38,39,40,41]. Measurements were performed prior to drug i.p. administration (baseline conditions) and 2 h after CBD injection. First, we tested a 10 mg/kg dose of CBD at Day 21 post MIA followed by a dose of 50 mg/kg CBD at Day 28 post MIA. In the following experiment, at Day 21 post MIA and 1.5 h after CBD administration, rats received the TRPV1 and TRPA1 antagonists SB-366791 (1 mg/kg) and AP-18 (0.2 mg/kg), respectively, whereas at Day 28 post MIA, animals receiving CBD were simultaneously coadministered the PPARγ antagonist GW9662 (2 mg/kg) or the TRPV4 antagonist HC-067047 (10 mg/kg) for 30 min before the assessment (similar to TRPV1 and TRPA1 antagonists). Control (intact) and MIA rats received the corresponding volume of vehicle solution (Kolliphor^®^ EL and 5% ethanol in 0.9% saline) either in one or two injections. Control, MIA, and CBD rats received the same treatment at both Days 21 and 28 post MIA. In the case of antagonist cotreatment, HC-067047 was administered to rats that had previously received AP-18, whereas GW9662 was administered to rats that had previously received SB-366791. Baseline measurements were performed 24 h before drug treatment, while drug effects were assessed 2 h after CBD i.p. administration. The investigator performing behavioral experiments was blinded to the treatment groups.

### 4.7. Kinetic Weight Bearing

To characterize pain behavior in the MIA model, we used kinetic weight bearing (KWB), a novel instrument developed by Bioseb (France). Sensors placed on the ground measure weight borne by each individual paw during the walking sequence of a freely moving animal, while a built-in camera detects body shape and the center of gravity of the animal, which is then used for further analysis. The rats were trained for a week to move through a corridor (50 cm × 130 cm) before the actual experiment. Measurements were made on D21 and D28 following MIA administration. Data collection was terminated when 3 validated runs were obtained or after 5 min of acquisition. All collected runs for each animal were then averaged for further statistical analysis. If the animal did not run during this time window, it was excluded from further analysis; thus, the number of samples for KWB may vary. All the recorded data were then validated and refined by a blinded observer, who carefully examined video recordings and verified that the animal was not stopping during the run or that the detected signal was ascribed to a proper paw.

### 4.8. Hargreaves Test

Animals were placed in Plexiglas boxes on top of a glass surface. After 5 min of habituation, a heat source was applied on the plantar surface of the right hind paw, and the latency of paw withdrawal from the heat stimulus was recorded. Withdrawal paw latency (heat sensitivity) was obtained using an average of three measurements recorded. Animals underwent three habituation sessions of 5 min each prior to testing sessions. To prevent tissue damage, the cutoff latency was set at 20 s.

### 4.9. Cold Plate Test

Cold hyperalgesia was assessed using a hot/cold plate (Model 35100, Ugo Basile) [42]. Rats were placed on a cold stainless steel plate maintained at 4 °C, and the number of lifts for each hind paw was counted for 120 s to prevent tissue damage.

### 4.10. Von Frey Test

For the assessment of mechanical allodynia, calibrated von Frey monofilaments (Bioseb, France) were used. Rats were placed in Plexiglas cages with a wire net floor 5 min before the experiment. Von Frey filaments were applied to the mid plantar surface of the ipsilateral hind paw according to the up and down method [43,44]. Each filament was applied thrice for an approximately 2- to 3-s period or until a withdrawal response was evoked. After response, the paw was retested with monofilaments in descending order until no response occurred, at which point monofilaments were again applied in ascending order until the response could once again be evoked. The monofilament that evoked the final reflex was noted as the paw withdrawal latency. The strength of the von Frey monofilament bending forces was as follows: 0.4; 0.6; 1.0; 1.4; 2; 4; 6; 8; 10; 15 and 26 g as a cutoff for response.

### 4.11. RNA Preparation

Tissue was dissected on Day 28 after MIA administration and 5 h after the last drug administration. We selected half of the dorsal lumbar spinal cord ipsilateral to the damaged knee and the superficial part of the femoral condylar cartilage (~5 mm) for isolation in reference to our previous studies [2,3]. Tissue was harvested and placed on ice in RNAlater and then stored at −80 °C. Extraction of high-quality RNA from cartilage and subchondral bone was performed according to a protocol published by Le Bleu et al., 2017. Briefly, the tissue was placed in 500 µL of TRIzol reagent (Invitrogen, Carlsbad, CA, USA). It was homogenized in a tissue lyser (Qiagen Inc., Hilden, Germany) at maximal frequency for a total of 5 min. Then, 500 µL of TRIzol was added to the Eppendorf tube followed by centrifugation at 12,000× *g* for 5 min at 4 °C to pellet undigested tissue. The supernatant was transferred to a new tube and 200 µL of chloroform was added. The sample was mixed by vigorous shaking for 30 s, allowed to stand for 3 min at room temperature, and then centrifuged at 12,000× *g* for 15 min at 4 °C. The aqueous layer was transferred to a new tube, and a mixture of concentrated sodium chloride and sodium acetate was added to achieve final concentrations of 1.2 M and 0.8 M, respectively. RNA was then precipitated by the addition of 0.3 volume of 100% isopropanol followed by a 10-min incubation at room temperature. Centrifugation at 12,000× *g* for 10 min at 4 °C facilitated pelleting of the RNA precipitate, which was washed twice using 1 mL of 75% (V/V) ethanol, dried for 5–10 min at 37 °C, and reconstituted in RNase-free water. RNA was denatured for 12 min at 65 °C. RNA from the spinal cord tissue was isolated with a Total RNA Mini kit according to the manufacturer’s protocol (A&A Biotechnology, Gdańsk, Poland) with extra centrifugation at 12,000× *g* for 5 min at a 4 °C step from the abovementioned protocol to minimize lipid contamination. The RNA concentration was measured using a NanoDrop ND-100 Spectrometer (Thermo Scientific, Wilmington, DE, USA). Total RNA (1.5 μg) was converted to double-stranded cDNA using iScript Reverse Transcription Supermix (Bio-Rad, Hercules, CA, USA) according to the manufacturer’s protocol in a 20 µL total volume. The complete reaction mix was incubated in a thermal cycler according to the manufacturer’s protocol. cDNA was stored in −20 °C.

### 4.12. Quantitative Polymerase Chain Reaction (qPCR)

The reaction was performed on Hard-Shell, thin wall PCR plates (Bio–Rad, Hercules, CA, USA, #HSP0601) with TaqMan probes and TaqMan Universal PCR Super Mix (Bio-Rad, Hercules, CA, USA) in a thermocycler C1000™ CFX96™ Real-Time system (Bio-Rad, Hercules, CA, USA) according to the manufacturer’s protocol as follows: denaturation for 30 s at 95 °C; followed by 40 cycles of denaturation for 5 s at 95 °C, annealing, extension and plate reading; then, 30 s at 60 °C. Twenty microliters of cDNA was diluted in 80 µL of RNase-free water. Then, 5.5 µL of cDNA solution was added to 4.5 µL of TaqMan Universal PCR Super Mix working solution. Samples were not run in duplicates. The threshold cycle (CT) value (cycle during which the fluorescence exceeds the threshold value) for each gene was normalized to the CT value of the beta-2 microglobulin (*B2m*) reference gene, which was selected based on literature findings [45,46]. RNA abundance was calculated as 2−(normalized ΔCt). The results are presented as the fold change proportional to the expression level in intact animals. The following assays (TaqMan Gene Expression Assays, Life Technologies, Carlsbad, USA) were used in the experiment: Rn00560865_m1 (*B2m*), Rn00563255_m1 (*Comp*), Rn00580555_m1 (*Ccl2*), Rn01410330_m1 (*Il6*), Rn00577366_m1 (*Fabp3*), Rn04342831_s1 (*Cb2*), Rn00583117_m1 (Trpv1), Rn00577086_m1 (*Faah*), Rn00440861_m1 (Prkcg), Rn01432300_g1 (*Prkaca*), Rn00578842_m1 (*Mapk14*), Rn00820922_g1 (*Mapk3*), Rn01483828_m1 (*Ptgs2*), Rn01461082_mL (*Alox12*), Rn01473803_m1 (*Trpa1*), Rn00576745_m1 (*Trpv4*), Rn00592665_m1 (*Trpm8*), Rn01482210_m1 (*Htr3a*), Rn00568803_m1 (*Gabra5*), and Rn00440945_m1 (*Pparg*), Rn03037213_s1 (*Gpr55*).

### 4.13. Statistical Analysis

The analysis was performed using Prism V.5 (GraphPad Software). Data were first examined for Gaussian distribution by the Shapiro–Wilk normality test and the equality of variances by the Brown–Forsythe test. Normally distributed data with equal variances were analyzed using ANOVA with Tukey’s post hoc test for comparison of the treatment effects. In case of inequal variances, data were analyzed using Welch and Brown–Forsythe ANOVA and Dunnett’s T3 multiple comparison test. Von Frey data did not meet the criteria for normal distribution; therefore, they were analyzed with the Kruskal–Wallis test followed by Dunn’s multiple comparisons test. The number of animals used in specific experiments is denoted under the graphs. Outlier values above/below the mean ± 2 * standard deviation were excluded from the analysis. The data were considered significant only when *p* < 0.05.

## 5. Conclusions

**Figure 11:**
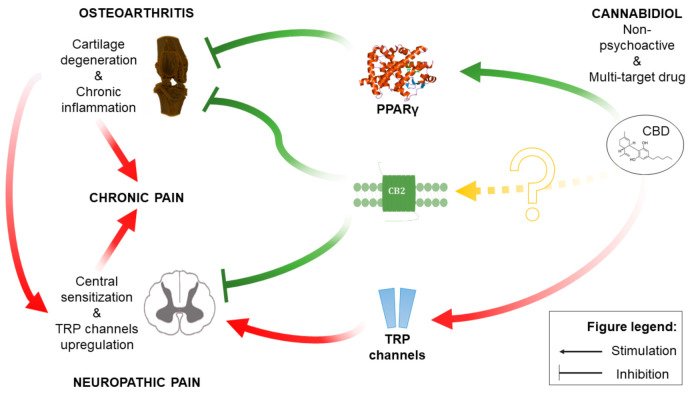
Schematic representation of the CBD mechanism of action within OA pathophysiology.

Herein, the presented results reveal the complex mechanism of CBD action in OA. We have identified the most relevant molecular targets of CBD and the spinal cord as the main site of its action. Our results highlight the importance of molecular changes regarding pain pathway sensitization occurring in OA as a confounding factor in the analgesic efficacy of drugs. We identified the PPARγ receptor as the most influential target for beneficial CBD effects in OA. On the other hand, we uncovered that TRP channel upregulation in OA and activation by CBD is a significant disruptive factor for CBD analgesic effects (see Figure 11 for the schematic representation of key factors in the CBD mechanism of action). Our results pave the way for novel CBD-based therapy for OA with improved therapeutic potential but also encourage the use of bioinformatic tools to predict the CBD mechanism of action in different conditions.

## Figures and Tables

**Table 1:** Biological processes involving CBD molecular targets associated with neuropathic pain and osteoarthritis. The results were obtained in STRING with Gene Ontology using the whole genome as a background.

Biological Processes	Protein Names	*p* Value	False Discovery Rate
thermoception	TRPA1, TRPM8, TRPV1	3.36 × 10^−9^	3.20 × 10^−6^
response to temperature stimulus	TRPA1, PPARG, TRPM8, TRPV4, TRPV1	8.35 × 10^−9^	3.98 × 10^−6^
ion transport	TRPA1, PPARG, TRPM8, GABRA5, HTR3A, TRPV4, TRPV1	5.60 × 10^−7^	1.30 × 10^−4^
cellular calcium ion homeostasis	TRPA1, TRPM8, GPR55, TRPV4, TRPV1	7.37 × 10^−7^	1.40 × 10^−4^
calcium ion transmembrane transport	TRPA1, TRPM8, TRPV4, TRPV1	1.84 × 10^−6^	1.90 × 10^−4^
response to cold	TRPA1, PPARG, TRPM8	1.97 × 10^−6^	1.90 × 10^−4^
detection of chemical stimulus involved in sensory perception of pain	TRPA1, TRPV1	3.52 × 10^−6^	2.50 × 10^−4^
chemical homeostasis	TRPA1, PPARG, TRPM8, GPR55, TRPV4, TRPV1	3.10 × 10^−6^	2.50 × 10^−4^
cannabinoid signaling pathway	CNR2, GPR55, FAAH	3.52 × 10^−6^	2.50 × 10^−4^
ion transmembrane transport	TRPA1, TRPM8, GABRA5, HTR3A, TRPV4, TRPV1	3.10 × 10^−6^	2.50 × 10^−4^

**Table 2:** Impact of specific molecular targets of CBDs on the protein–protein interaction network in the neuropathic component of OA. The vertex degree is the number of edges that are incident to the node, which represents the number of interactions for specific proteins in the whole protein–protein interaction network associated with the diseases. Pharmacodynamic details were obtained from the cited literature.

Target	Vertex Degree	Activity (nM)	Interaction	Source
PPARG	572	100	full agonist	[6]
CNR2	294	34	partial agonist	[7]
GPR55	280	445	antagonist	[8]
TRPV1	274	1000	full agonist	[9]
HTR3A	194	329	negative allosteric modulator	[10]
TRPA1	148	110	full agonist	[9]
TRPV4	120	800	full agonist	[9]
GABRA5	100	1400	positive allosteric modulator	[11]
TRPM8	92	60	antagonist	[9]
FAAH	80	1520	inhibitor	[9]

## Data Availability

Experimental data may be made available by the authors upon request. Data regarding CBD pharmacodynamics as well as software used for analysis are available at https://jakub-mlost.shinyapps.io/CBDpharm/ or https://github.com/JakubMlost/CBDpharm (accessed on 21 September 2021).

## Supplementary Materials

The following are available online at https://www.mdpi.com/article/10.3390/ph14100964/s1, Table S1: A database of CBD molecular targets.
